# The Efficacy of Cognitive Remediation Therapy for Anorexia Nervosa: A Systematic Review of Systematic Reviews

**DOI:** 10.3390/brainsci14020118

**Published:** 2024-01-24

**Authors:** Gloria Marchesi, Davide Maria Cammisuli, Michelle Semonella, Gianluca Castelnuovo, Giada Pietrabissa

**Affiliations:** 1 Department of Psychology, Catholic University of Milan, 20123 Milan, Italy; gloria.marchesi03@icatt.it (G.M.); davide.cammisuli1@unicatt.it (D.M.C.); gianluca.castelnuovo@unicatt.it (G.C.); 2 Department of Psychology, Bar-Ilan University, Ramat Gan 590002, Israel; michelle.semonella@biu.ac.il; 3 Clinical Psychology Research Laboratory, I.R.C.C.S. Istituto Auxologico Italiano, 20149 Milan, Italy

**Keywords:** cognitive remediation therapy, anorexia nervosa, systematic review, clinical psychology

## Abstract

Background: Cognitive remediation therapy (CRT) for anorexia nervosa (AN) is an intervention specifically focused on addressing cognitive difficulties associated with the eating disorder. This systematic review of systematic reviews and meta-analysis aimed to provide a summary of the existing literature examining the efficacy of CRT in improving the neuropsychological, psychological, and clinical parameters of patients with AN. Methods: Systematic reviews and meta-analyses were sought in electronic databases, encompassing studies that explored the impact of CRT on AN. Three eligible reviews were identified based on the inclusion criteria. The Revised Assessment of Multiple SysTemAtic Reviews (R-AMSTAR) was employed to evaluate the methodological quality of the reviews, and all included reviews demonstrated satisfactory methodological quality with an R-AMSTAR score of ≥22. Relevant information was extracted from each review and qualitatively compiled. Results: Findings suggest that CRT can help people increase their awareness of cognitive styles and information processing and have a positive effect on patients’ responses to treatment. Conclusions: Further research is required to better understand its impact on other relevant outcomes, including psychological variables, to optimize the treatment’s benefits.

## 1. Introduction

Anorexia Nervosa (AN) is a severe and persistent eating disorder (ED) characterized by self-starvation, a reduction in body weight, and the perception of distorted body size and shape [1]. It is the leading cause of death among all mental health conditions, and an estimated 0.9% of women and 0.3% of men will experience AN at some point in their lifetime [2]. The available evidence for treating AN is limited [3,4] due to patients’ uncertainty toward change, drop-out rates of 20–40% [5], and low treatment acceptance [6].

In terms of clinical presentation, people with AN often focus on specific behaviors and routines, such as rigid rules [7]. Consequently, it is crucial to devise interventions that target the risk and maintenance aspects of AN rather than the core symptoms and concerns of the ED [8,9]. 

Particularly, neuropsychological research has highlighted two main cognitive deficits in AN, i.e., cognitive inflexibility (or limited set-shifting) [10] and intense attention to detail (or reduced central coherence) that indicate reduced executive and visual-constructive functions [11,12,13]. Reduced set-shifting refers to cognitive difficulties in shifting attentional control upon different tasks and in using different cognitive strategies as environmental circumstances change, while reducing central coherence involves intense focus on details rather than thinking in a holistic processing of information [14,15,16]. Among individuals with AN, both weak set-shifting and reduced central coherence are typically observed through obsessions with food, body image, weight, and compulsive actions like monitoring calories and exercising. Both set-shifting and weak central coherence could contribute to the development of AN [17] and could persist despite weight gain [18], thus contributing to diminished participation and compliance with treatment [19].

The difficulty of addressing AN has led to the conceptualization of novel approaches aimed at addressing these challenges, therefore fostering patients’ awareness of their cognitive styles and encouraging the adoption of alternative strategies. 

In recent years, the evidence supporting cognitive remediation therapy (CRT) for AN has been growing [20,21]. CRT is a structured intervention delivered by paper-and-pencil or computer-based cognitive exercises centered on improving cognitive flexibility and holistic thinking. It directs its attention toward cognitive processes rather than emotional aspects, thus addressing cognitive function defects rather than the psychopathology of ED [22,23]. It is also aimed at increasing individuals’ motivation to change and overall quality of life [14,24,25,26,27,28,29,30].

However, the optimal use of CRT for AN treatment remains under exploration, and published systematic reviews and meta-analyses of CRT for AN reveal conflicting conclusions. 

The present study aims to provide a summary of published systematic reviews and meta-analyses on the efficacy of CRT in improving cognitive flexibility, central coherence, set-shifting ability, motivation to change, and the quality of life in patients with anorexia nervosa. 

## 2. Materials and Methods

This work was carried out following the Preferred Reporting Items for Systematic Reviews and Meta-Analyses (PRISMA) guidelines [31]. The protocol for this study was registered with the International Prospective Register of Systematic Reviews (PROSPERO), nr. CRD42023459389. Trial Registration: PROSPERO ID CRD4202345938.

### 2.1. Literature Search

A systematic literature search of six databases (Scopus, PubMed, Google Scholar, PsychINFO, the Cochrane Library, and the Centre for Review and Dissemination—CRD) was conducted between 4 September and 31 October 2023. Under the PICO framework (Patient problem or population; Intervention; Comparison or control and Outcome) [32], the search strategies included the following terms: (anorexia) AND (cognitive remediation therapy) AND (flexibility OR central coherence OR set shifting OR body mass index-BMI) OR eating disorder OR motivation OR quality of life). Search terms were systematically combined by Boolean and truncation operators. The search syntax was modified as appropriate for each database.

### 2.2. Inclusion and Exclusion Criteria

Only systematic reviews and meta-analyses that met the following criteria were included: (1) people with AN, (2) explored the efficacy of cognitive remediation therapy in at least one cognitive/psychological domain (e.g., cognitive flexibility, central coherence, set-shifting ability, motivation to change, and quality of life), and (3) received a methodological quality score of 22 or higher in the Revised Assessment of Multiple SysTemAtic Reviews (R-AMSTAR) [33]. Studies were excluded if they (1) considered only biomedical outcomes. No limitations were set for language and year of publication, or for the age, gender, and ethnicity of the sample.

### 2.3. Selection Process

Two reviewers (G.M. and G.P.) independently screened the eligibility of the articles based on their titles, abstracts, and then their full texts. Disagreements were solved through discussion with a third researcher (D.M.C.). Furthermore, reference lists were manually examined for the possible inclusion of relevant records. The review team included at least one person with methodological expertise in conducting systematic reviews (G.P. and M.S.) and at least two experts on the topic under review (authors G.C. and D.M.C.) [34]. 

**Figure 1:**
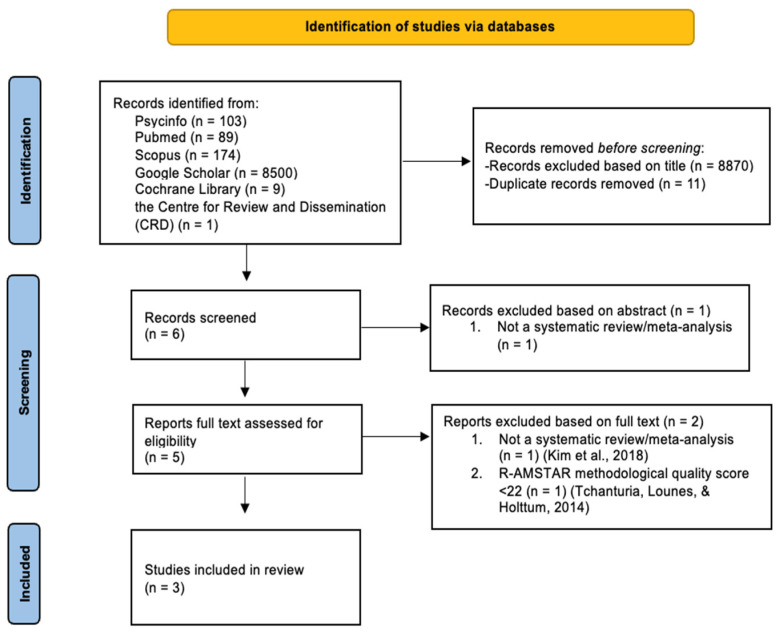
The PRISMA flowchart [36,37].

Following the PRISMA guidelines [35], the flowchart presented in Figure 1 provides step-by-step details of the study selection procedure.

### 2.4. Data Extraction

The following data were independently extracted by reviewers GM and GP, and any disagreements were resolved by consensus and consultation with a third researcher (author DMC): the author and year of publication, country, the aim of the review, the number of relevant included studies, study design, sample size, the age and gender of the participants, the duration of the intervention, format, and outcomes (Table 1).

The extracted data were used to produce a narrative summary of the effects of CRT on improving the outcomes of people with AN.

### 2.5. The Assessment of the Risk of Bias 

The R-AMSTAR checklist [33] was used to assess the methodological quality of the included systematic reviews and meta-analyses. It assesses the presence of 11 domains: 1. a priori design, 2. duplicate study selection and data extraction, 3. comprehensive literature exploration, 4. the incorporation of publication status as an inclusion criterion, 5. a list of included/excluded studies, 6. the attributes of included studies, 7. the evaluation of the scientific quality of selected studies, 8. the appropriate utilization of scientific quality in formulating conclusions, 9. the appropriate use of methodologies to combine study results, 10. the assessment of the potential publication bias, and 11. the inclusion of conflicts of interest. The score for each domain varies from 1 to 4, and the R-AMSTAR’s total scores range from 11 to 44. To include the reviews, a total score of 22 was mandatory. Two reviewers (G.M. and G.P.) evaluated the methodological quality of the selected reviews, and disagreements were resolved by a third researcher (D.M.C.) (Table 2).

### 2.6. Data Analysis and Synthesis

Initially, reviews were examined, and pertinent details were extracted and documented. The outcomes from various reviews were combined using a qualitative summary to inform the efficacy of the treatment. A summary of quantitative results was not calculated from the meta-analyses that incorporated comparable studies due to the limitation that a meta-analysis of meta-analyses can only be conducted if the data from individual studies are not duplicated across multiple meta-analyses [34,39]. Indeed, seven [40,41,42,43,44,45,46] out of the 29 studies reviewed were included in more than one selected systematic review and meta-analysis (see Appendix A).

## 3. Results

### 3.1. Study Selection

A total of 8876 articles were initially identified: 11 were duplicates while 8871 records were excluded by reading their title and abstract. The full text of the remaining five articles was then screened, resulting in the exclusion of two records for the following reasons: (1) not a systematic review/meta-analysis [36] and (2) R-AMSTAR methodological quality score < 22 [37].

Three studies presenting a summary of evidence on the efficacy of CRT for AN entered this second-order review: one study aimed at conducting a systematic review of single case reports, case series, and RCTs [38]; Tchanturia et al., (2017) carried out a systematic review of single or multiple case studies and qualitative contributions [21], and a systematic review and meta-analysis of RCTs was conducted by Hagan et al. 2020 [20].

### 3.2. The Characteristics of the Included Studies

Table 1 provides a summary of the characteristics of each included review. All of the reviews examined the efficacy of CRT for patients with AN.

The total number of participants across reviews ranged from 303 [20] to 367 [21]. In terms of gender, two studies [20,38] included a higher proportion of female participants, while one study reported no gender information [21]. None of the selected contributions set any age-related inclusion criteria, and the ages of the participants ranged from 12 to 62 years in the reviews. 

CRT treatments were delivered either individually or in groups, with a frequency ranging from one to three times per week and a total number of sessions ranging from 4 to 36. 

In addition, the review by Dahlgren et al., (2014) also included studies providing family interventions and computer-assisted CRT [38].

### 3.3. The Methodological Quality of Included Reviews

The R-AMSTAR scores of the three included systematic reviews and meta-analyses (see Table 2) ranged from 25 [21,38] to 32 [20], with a mean score of 27.33 (SD = 4.04). The highest scores were assigned to Item #3 (Was a comprehensive literature search performed?) and Item #6 (Were the characteristics of the included studies provided?), in which all three systematic reviews and meta-analyses achieved the maximum. 

Maximum scores were also obtained by Dahlgren et al., (2014) [38] in Item #5 (Was a list of studies—included and excluded—provided?), and by Hagan et al., (2020) [20] in Item #2 (Was there a duplicate study selection and data extraction?). In addition, minimum scores were given to Dahlgren et al., (2014) [38] and Tchanturia et al., (2017) [21] in Item #7 (Was the scientific quality of the included studies assessed and documented?) and Item #8 (Was the scientific quality of the included studies used appropriately in formulating conclusions?). Furthermore, Dahlgren et al., (2014) [38] received minimum scores in Item #2, Item #9 (Were the methods used to combine the findings of studies appropriate?), and Item #10 (Was the likelihood of publication bias assessed?), while Tchanturia et al., (2017) [21] received minimum scores in Item #4 (Was the status of publication—i.e., grey literature—used as an inclusion criterion?) and Item #11 (Was the conflict of interest included?). The highest scores across the selected systematic reviews and meta-analyses were for performing a comprehensive literature search (Item #3), and for providing the characteristics of the included studies (Item #6), the lowest scores were for documenting the scientific quality of the included studies (Item #7), and appropriately using the scientific quality of the included studies to formulate conclusions (Item #8). 

### 3.4. Single Cases

Single-case studies focused on patients’ experiences with CRT. Taken together, results showed the potential of CRT in enhancing patients’ understanding of their thinking styles, regardless of the improvement in outcomes. For example, one of the included studies [47] reported no significant post-treatment changes in the participant’s neuropsychological profile. Still, the subject provided positive feedback and suggested that she had gained more awareness and self-reflection on her cognitive patterns. Furthermore, the respondent showed stable weight and decreased AN symptomatology at a 7-month follow-up. 

### 3.5. Case Series

Different types of case series were reviewed. Studies [46,48,49,50,51,52] documented the applicability of CRT either in individual or group settings. Results also showed that CRT can be applied to patients with AN across ages and stages of the disease. 

Case series studies, including the pre- and post-quantitative assessments of outcomes, were difficult to compare as they largely varied in terms of the age of the participants, treatment intensity, and assessment measures. However, patients received approximately the same number of CRT sessions [53,54,55]. The results showed a decrease after CRT treatment in depression and significant positive changes in the attention span among adults, but not in adolescents. For example, in a pre-post study, Dahlgren, et al., (2013) found significant changes in visuospatial memory and both processing and verbal fluency, but not in the executive functioning domain [46]. In another pre-post study [47], adolescent inpatients receiving CRT reported improvements in cognitive flexibility after the intervention, while their healthy counterparts who didn’t receive the intervention did not. No significant improvements were, instead, reported in central coherence. A subsequent follow-up study employing the same sample, together with additional participants, compared subjects with AN who received CRT with those who received no additional treatment [42]. Data were collected at the beginning and end of the intervention, as well as 6 months after treatment termination. No significant changes in neuropsychological and clinical measures were reported at follow-up, although the BMI increase in the CRT sample showed a noteworthy tendency. 

A three-group uncontrolled trial comparing patients with severe AN receiving CRT to a group of patients receiving treatment as usual (TAU) plus CRT and a TAU condition showed that only participants assigned to the CRT groups had a post-treatment improvement in motivation to recover, set-shifting, central coherence, and switching task abilities [28].

Last, one-group pre-post studies observed significant improvements in set-shifting among patients with AN receiving CRT [44,56,57,58]. Still, while in Tchanturia et al., (2007) [57], self-reported flexibility was significantly higher post-CRT, no significant changes were reported in self-esteem or cognitive flexibility by Genders & Tchanturia (2010) [58].

### 3.6. Randomized Control Trials (RCTs)

Included RCTs [26,40,41,42,43,59] either reported significant changes in set-shifting and central coherence at the end of the CRT intervention or revealed non-significant improvements in the above-mentioned variables, depending on the outcome measures.

Specifically, an RCT comparing CRT and cognitive behavioral therapy (CBT) [43] showed increased set-shifting and central coherence abilities in patients receiving CRT, as well as lower dropout rates than their CBT counterparts. Still, no significant between-group differences were found for weight, BMI, or ED symptoms. Other RCTs [40,41], comparing CRT with nonspecific neurocognitive therapy (NNT) or TAU, found significant changes in cognitive flexibility and the quality of life related to ED in the CRT group. Dropout rates, instead, did not vary significantly, and no between-group differences were reported for BMI.

A single RCT [45] revealed improvements in caloric intake and eating-related anxiety in patients receiving exposure and response prevention therapy for AN (AN-EXRP) compared to the CRT group. 

No pre-post within-group differences were found in anxiety, depression, obsessive-compulsive symptoms, or perfectionism in CRT groups [41,42,59]. 

### 3.7. Qualitative Studies

A few of the case studies included qualitative assessments of patients’ feedback, but only a single investigation was specifically intended to examine patient feedback letters after individual CRT [60]. Most of the participants reported a positive involvement in CRT and expressed that they had acquired knowledge about cognitive patterns and problem-solving techniques that could be used in everyday situations.

## 4. Discussion

To the best of our knowledge, this work represents the first attempt to summarize the state of the art on the efficacy of CRT for AN by conducting a systematic review of systematic reviews and meta-analyses on the topic. Throughout the selected systematic reviews and meta-analyses, the investigations consisted of RCTs, single case studies, case series, and qualitative evaluations. Inevitably, differences in methodology, delivery modality and setting, and outcome measures across studies posed important challenges in comparing and generalizing research findings. Still, these results have the potential to provide practical insight into the context of the implementation of CRT solutions for AN.

Specifically, regarding neuropsychological outcomes, results were contradictory for both central coherence and set-shifting abilities, the main cognitive domains targeted in the CRT intervention. Several studies found small to moderate improvements in central coherence among patients with AN who received CRT, while others reported no changes [41,42,43]. Also, small-to-moderate and moderate-to-large improvements in set-shifting abilities following CRT were observed, irrespective of the outcome measure [41,43]. 

Moreover, cognitive flexibility and executive functioning improved significantly following CRT across studies. However, the absence of further changes in cognitive flexibility after 6 months from treatment termination observed in Herbrich et al., (2017) suggests the need for a further exploration of the short- and long-term effects of CRT on neurocognitive domains [42].

Regarding psychological outcomes, Dingemans et al., (2014) [41], Herbrich et al., (2017) [42], and Lock et al., (2018) [59], found no significant differences in anxiety and depression symptoms at the end of the treatment in patients with AN undergoing CRT compared to patients who received TAU. Conversely, Dahlgren et al., (2013) [46] and Tchanturia et al., (2008) [54] identified a decrease in depressive symptoms in those who received CRT in their pre- and post-investigations. Furthermore, Diengemans et al., (2014) [41] and van Passel et al., (2020) [61] noted small to moderate improvements in ED-related quality of life. More research is needed to clarify these conflicting findings and investigate the influence of these comorbidities as moderators. Finally, no significant differences were detected in ED symptoms, both among adults and adolescents. Notably, Lock et al., (2013) [43] showed that CRT is not superior to CBT in alleviating symptoms related to ED, but it has an important effect on certain neurocognitive functions including cognitive flexibility and executive functioning.

Overall, these results point out the specific effect that CRT has on improving the neuropsychological functioning of patients with AN. Its effectiveness in enhancing the psychological well-being of such individuals, on the other hand, remains questionable—at least in the short term. This is not surprising, given the nature, characteristics, and goals of the intervention, which may represent an alternative-integrative approach to treatments focused on managing individuals’ emotional difficulties (e.g., CBT).

It is also interesting to note that, regardless of the statistical significance of the outcomes of the intervention, qualitative studies on the topic agree in suggesting that CRT is useful in promoting the understanding of AN patients about their thinking patterns and in enabling them to learn problem-solving techniques crucial for the autonomous management of their daily difficulties. Consistently, post-treatment improvements in motivation to change were observed. Increased awareness of one’s active role in problem resolution coupled with enhanced perceived self-efficacy, may have contributed to boosting individuals’ intrinsic motivation for treatment.

These considerations lead to the hypothesis that post-CRT, statistically significant improvements in neurological, psychological, and clinical outcomes are expected more in the long term than in the short term, as a result of the person’s continuous commitment to change. Studies with follow-ups extending beyond 12 months from the end of CRT treatment should be implemented to test this possibility. 

Regarding clinical outcomes, most of the included studies did not report significant between-group differences in BMI and weight in individuals who received CRT treatment compared to TAU, but BMI decreased among those receiving CRT as reported in case studies. While weight gain is an expected outcome for individuals with AN participating in standard treatments with or without CRT integration, improved clinical parameters recognized by case studies reinforce the role of CRT in determining such an improvement. 

Moreover, considering treatment delivery, individual CRT showed lower dropout rates and fostered a more positive patient-therapist alliance compared to group CRT [38]. This might be due to the fact that tasks assigned during group CRT were not perceived to be as relevant as in individual therapy [42,44], therefore they did not strengthen individuals’ motivation to change.

## 5. Strengths and Limitations

A notable strength of this contribution lies in the rigor of our data extraction and analysis process, which involved multiple researchers independently screening and scrutinizing the information, and the evaluation of the methodological quality of the included systematic reviews and meta-analyses. However, certain limitations must be considered. First, the variety in study design, sample characteristics (such as sample size and age, whether they were inpatients or outpatients), intervention methods (such as individual, family, or group therapy), treatment intensity, and the choice of outcome measures made it challenging to compare findings from the selected reviews and draw solid conclusions about the efficacy of CRT. Also, methodological flows and potential biases of the included studies as assessed by the R-AMSTAR checklist might have influenced the conclusions of the present work.

## 6. Suggestions and Implications for Future Research

The findings from this systematic review of systematic reviews and meta-analyses indicate that there is a need to implement research in CRT. First, future investigations aimed at testing and/or comparing different ways to deliver CRT should be carried out to better understand their specific advantages and disadvantages, and their effectiveness in improving cognitive, psychological, and clinical outcomes. This might include the testing of differences between individual or group therapy, but also the impact of computer-assisted CRT (CA-CRT) sessions on patients’ outcomes. Indeed, according to Brockmeyer (2013), CA-CRT might lead to increased awareness and the development of new thinking strategies but more studies should be conducted on the topic [40].

Moreover, the single report published on family CRT [62], showed a positive impact of the intervention in enhancing the understanding of how cognitive styles influence family dynamics and improving communication and cooperation among family members during treatments. Family CRT should, therefore, be further explored, also in integration with other therapeutic approaches.

Due to variations in outcome measures among the included records, the generalization of conclusions is complex, and future research might also consider employing more consistent outcome measures—especially concerning psychological outcomes.

Indeed, besides the fact that CRT mainly focuses on improving cognitive functioning, the evaluation of the impact that the intervention might have on emotional aspects is also relevant in future studies, which might also consider implementing and testing a specific CRT module about emotions. 

Similarly, research comparing CRT with other treatment methods is limited, like the testing of the integrative impact of CRT on target outcomes. A broader range of comparative-integrative studies should, therefore, be further developed to help determine the role of CRT in the global treatment plan. 

Moreover, since studies mainly focus on short-term effects, longitudinal studies would be crucial for understanding the long-term benefits of CRT on AN.

Overall, the selected studies support the feasibility of CRT across different ages and severity levels. However, it was not possible to explore how different types of patients respond to CRT. Future studies could focus more on individual differences, including the impact of age, severity of the disorder, and other relevant psychological and medical conditions on the effectiveness of CRT.

Specifically, knowledge is limited regarding the efficacy of CRT for adolescents diagnosed with AN. Since early engagement in treatment is associated with better outcomes [21], future studies should focus more on this age group.

## 7. Conclusions

Despite the findings from this systematic review of systematic reviews and meta-analyses revealing limited data, the present study suggests that CRT can help patients with AN become more aware of their cognitive styles and information processing and have a positive impact on treatment response. Some issues persist about CRT arrangements in regards to the intensity and duration needed to improve central coherence and set-shifting. Further research is needed to better understand CRT effects and how to maximize its unique benefits for the treatment of AN.

## Figures and Tables

**Table 1:** Characteristics of included studies.

First Author (Year)	Country	Aim of the Review	Included Studies	Study Design	Sample Size (n)	Age (Year): Mean (SD) or Range	Women: n or %
Dahlgren (2014) [38]	UK	To systematically review studies of CRT for AN and to discuss findings with references to clinical practice and future research	21	Single or multiple case studies and RCT	362	12–62	355
Hagan (2020) [20]	USA	To determine the effect of CRT for AN in comparison with control treatments in RCTs on neuropsychological deficits at the end of the treatment and to assess the effect of CRT for AN on dropout, related eating disorders, and other psychological outcomes at the end of the treatment	9	RCT	303	22.67 (0.37)	93.9%
Tchanturia (2017) [21]	UK	To evaluate the evidence about the efficacy of CRT in children and adolescents with AN	9	Single or multiple case studies and qualitative assessments	367	14.97 (0.65)	NR
**First Author (Year)**	**N° of Sessions**	**Frequency of Sessions**	**Format**	**Clinical Outcome (Measure)**	**Psychological Outcome** **(Measure)**	**Neuropsychological Outcome ** **(Measure)**	
Dahlgren (2014) [38]	Between 4 and 30 session	From 1 to 3 times per week	Individual, groups, or systemic	BMI; Calorie intake	Depression; Patients’ experience with CRT; Treatment acceptance	Cognitive flexibility; Cognitive set-shifting; Perfectionism; Rigidity; Reflexive skills; Impulse regulation; Visuospatial memory; Working memory; Verbal fluency; Global information processing; Brixton & CatBat tasks; Executive function (BRIEF-SR)	
Hagan (2020) [20]	Between 6 and 36 sessions	From 1 to 3 times per week	Individual or groups	BMI; Range, frequency, and severity of behaviors associated with ED (EDE/EDEQ); Calories consumed during meal	Depression (BDI and DIKJ); Quality of life (EDQoL); Anxiety (STAI(C)-T), ED (ChEDEQ and YBC-EDS); Obsessive and compulsive symptoms (CY-BOCS)	Global processing ability, visuospatial ability, and visuospatial memory (RCFT); Executive functions (D-KEFS CWIT); Cognitive control related to the updating, representation, and maintenance of frequently changing task rules	
Tchanturia (2017) [21]	Between 8 and 10 sessions	NR	Individual or groups	NR	Identification and quantification of adaptation patterns (CWT); Aspects of personality and cognitive impairment (GEFT); Motivation (Motivational Ruler); Satisfaction (Satisfaction Questionnaire)	Global processing ability, visuospatial ability, and visuospatial memory (RCFT); Attention (TMT-4); Executive functions (BRIEF-SR, Tower Test, and D-KEFS); Neuropsychological functioning in AN (The Ravello Profile); Cognitive flexibility (CFS)	

Note. NR—not reported; AN—Anorexia Nervosa; BDI—Beck Depression Inventory; BMI—Body Mass Index; BRIEF-SR—Behavioral Rating Inventory for Executive Functioning-Self Report; CFS—Cognitive Flexibility Scale; ChEDEQ—Child Eating Disorder Examination Questionnaire; CRT—Cognitive Remediation Therapy; CTMT—Comprehensive Trail Making Test; CWT—Serial Colour-Word Test; CY-BOCS—Children’s Yale-Brown Obsessive-Compulsive Scale; DIKJ—Depression Inventory for Children and Adolescents; D-KEFS CWIT—Delis-Kaplan Executive Function System Color-Word Interference Test; ED—Eating disorder; EDE—Eating Disorder Examination; EDEQ—Eating Disorder Examination Questionnaire; EDQoL—Eating Disorder Quality of Life Questionnaire; GEFT—Group Embedded Figures Test; RCFT—Rey-Osterrieth Complex Figure Test; RCT—randomized control trial; STAI(C)-T—State-Trait Anxiety Inventory for Children; TMT-4—Trial Making Test Condition 4; UK—the United Kingdom; USA—the United States of America; WCST—Wisconsin Card Sorting Test; YBC-EDS—Yale-Brown-Cornell Eating Disorder Severity Scale.

**Table 2:** Article Quality Assessment.

First Author (Year)	Item 1	Item 2	Item 3	Item 4	Item 5	Item 6	Item 7	Item 8	Item 9	Item 10	Item 11	Total Score
Dahlgren (2014) [38]	2	1	4	3	4	4	1	1	1	1	3	25
Hagan (2020) [20]	2	4	4	2	2	4	3	3	2	3	3	32
Tchanturia (2014) [37]	2	1	4	3	1	4	1	1	1	1	2	21
Tchanturia (2017) [21]	2	3	4	1	2	4	1	1	3	3	1	25

Note. 1. Was an “a priori” design provided? 2. Was there duplicate study selection and data extraction? 3. Was a comprehensive literature search performed? 4. Was the status of publication (i.e., grey literature) used as an inclusion criterion? 5. Was a list of studies (included and excluded) provided? 6. Were the characteristics of the included studies provided? 7. Was the scientific quality of the included studies assessed and documented? 8. Was the scientific quality of the included studies used appropriately in formulating conclusions? 9. Were the methods used to combine the findings of the studies appropriate? 10. Was the likelihood of publication bias assessed? 11. Was the conflict of interest included?

## Data Availability

Data are available on request due to privacy and ethical restrictions.

## Appendix A. Target Studies Included in Each Selected Systematic Review/Meta-Analysis

	**Dahlgren (2014)** [38]	**Hagan (2020)** [20]	**Tchanturia (2017)** [21]
Abbate-Daga (2012) [53]	X		
Brockmeyer (2013) [40]	X	X	
Dahlgren (2013) [46]	X		X
Davies (2005) [63]	X		
Dingemans (2014) [41]	X	X	
Easter (2011) [52]	X		
Genders (2010) [58]	X		
Giombini (2016) [29]			X
Giombini (2017) [29]			X
Harrison (2018) [28]			X
Herbrich (2017) [42]		X	X
Lask (2013) [62]	X		
Lock (2013) [43]	X	X	
Lock (2018) [59]		X	
Pitt (2010) [56]	X		
Pretorius (2007) [64]	X		
Pretorius (2012) [44]	X		X
Sproch (2019) [26]		X	
Steinglass (2014) [45]	X	X	
Tchanturia (2006) [65]	X		
Tchanturia (2007) [57]	X		
Tchanturia (2008) [54]	X		
van Noort (2015) [47]			X
van Noort (2016) [66]			X
van Passel (2020) [61]		X	
Whitney (2008) [51]	X		
Wood (2011) [49]	X		
Zuchova (2013) [50]	X		
